# Effect of a Conditional Cash Transfer Program on Nutritional Knowledge and Food Practices among Caregivers of 3–5-Year-Old Left-Behind Children in the Rural Hunan Province

**DOI:** 10.3390/ijerph15030525

**Published:** 2018-03-15

**Authors:** Yefu Zhang, Meimei Ji, Jiaojiao Zou, Tong Yuan, Jing Deng, Lina Yang, Mingzhi Li, Hong Qin, Jihua Chen, Qian Lin

**Affiliations:** 1 Department of Nutrition Science and Food Hygiene, Xiangya School of Public Health, Central South University, 110 Xiangya Road, Changsha 410078, Hunan, China; yefuzhang@foxmail.com (Y.Z.); jimeimei1024@foxmail.com (M.J.); zjj170605@foxmail.com (J.Z.); yuantong168@foxmail.com (T.Y.); ylnly1997@csu.edu.cn (L.Y.); lmz1976@126.com (M.L.); qinhong@csu.edu.cn (H.Q.); 2 Department of Epidemiology and Statistical Science, Xiangya School of Public Health, Central South University, 110 Xiangya Road, Changsha 410078, Hunan, China; dengjing2@126.com

**Keywords:** left-behind children, caregiver, rural area, nutrition knowledge, food practice, China

## Abstract

Left-behind children (LBC) are a unique population in China, whose numbers have increased dramatically in recent years. Most caregivers of left-behind children (CLBC) are grandparents who lack knowledge about proper nutrition and food practice, putting LBC at greater risk for malnutrition. A cluster randomized controlled trial was carried to assess the effectiveness of the conditional cash transfer (CCT) program. Forty rural villages from Fenghuang County and Pingjiang County of Hunan province were selected. The villages were randomized into the intervention and control groups. In the intervention group, caregivers received a cash transfer conditional on bimonthly health education attendance, bringing LBC in for vaccinations, and on-time annual health checks. The control group received routine health services only. Two rounds of questionnaire surveys were conducted in March 2015 and July 2016. Questionnaires and in-person interviews were used to assess the changes in nutritional knowledge and food practices among CLBC. Among 447 valid subjects, CLBC in the intervention group were significantly more likely to correctly understand the importance of children’s height and weight measurements, food variety, inclusion of eggs and dairy in the diet, and anemia identification and prevention. Intervention group CLBC were also significantly more likely to prepare dairy products and eggs for their children. Generalized liner mixed model (GLMM) analysis showed that CLBC nutrition knowledge was improved significantly in the intervention group (adjusted *p* value = 0.01), and there were also positive changes in their food practice (adjusted *p* value = 0.047). This CCT intervention turned to be effective with respect to rural caregivers’ nutritional knowledge and food practice behavior. The findings from this project could be helpful for future health strategies targeting rural children, in particular the LBC group.

## 1. Introduction

Left-behind children (LBC) refer to children with at least one parent living in another area due to employment; consequently, the parents and children do not all live together at home [1]. Most LBC in China are concentrated in rural areas, particularly in Sichuan, Anhui, Henan, Guangdong, Hunan, Jiangxi and other central and southern provinces. In our study site of the Hunan Province there are 2.225 million LBC, accounting for 51.14% of all rural children in the province (2010) [2]. A product of China’s rapid economic and social transformation in recent decades, rural LBC are an especially vulnerable population and will remain so for a long period of time. 

National survey data show that the prevalence of stunting in children under the age of 5 in rural areas of China is 12.1%, while the prevalence of stunting in children left behind by mothers is 20–30% [3]. The Chinese government has implemented some successful nutrition programs targeting rural children under 2 years of age, and school age children. However, for 3–5-year-old left-behind children (which represent a blind spot in health intervention programs), undernutrition is particularly prominent. In China, most poor rural villages have no kindergarten or care centers. Equity in accessing health services and nutrition among rural preschool children has received little attention. The nutritional knowledge and food practice behaviors of caregivers of LBC (CLBC) directly affect children’s health. Our baseline data showed that about 75% of LBC are separated from both parents (with both parents having migrated) and that most of the caregivers are elderly grandparents (83%), or sometimes distant relatives or neighbors [4], who are less-educated and do not pay sufficient attention to dietary diversity, nutrition, and food sanitation. They have difficulties in providing children with a reasonable and nutritious meal supply. While the nutritional and health status of rural LBC aged 3–5 years is worthy of attention, there is presently no nutrition improvement policy for this particular group.

In recent years, the effectiveness of conditional cash transfers (CCTs) for improving nutrition and health status among low-income people has gradually come into focus [5,6]. CCTs are a form of social security in which beneficiaries must carry out certain actions (such as improving health, education, or nutrition levels) as a precondition for receiving cash subsidies, which reduce low-income families’ out-of-pocket expenditure [7,8]. The cash transfer has potential to increase the household’s purchasing power for better quality food in greater quantities, and to reduce transport costs for accessing public health services for LBC. Studies have shown that CCTs can raise the level of consumption of low-income households and increase household disposable income toward high-quality, high-nutritional content foods. A large, randomized controlled trial in Indonesia for two years showed that CCTs significantly increased food consumption, especially protein-rich foods such as milk and fish [9]. A 2013 systematic review of Brazil’s CCT program showed that the CCT intervention was positively associated with the recipient’s diet and nutrition [10]. Eeshani et al. emphasized that CCTs can promote the transformation of key parenting methods, including intake of high-protein food and seeking medical behavior, which help reduce child malnutrition [11]. Six CCT programs included health and nutrition education workshops (CCT programs in Brazil, Colombia, Mexico, Nicaragua, El Salvador, and Peru). The segmented nutrition education workshops were the most appropriate way for the main caregivers to learn about nutrition and health, which in turn could improve nutrition behaviors for young children under their care. We used a randomized controlled trial that combined nutrition education, primary health care, and cash subsidies [12]. The goal of this study was to assess the impact of CCT interventions on the nutrition knowledge and food practice behaviors of caregivers of LBC aged 3–5 years.

## 2. Methods

### 2.1. Trial Design

The data for this article was from a cluster randomized control trial to evaluate the effectiveness of a conditional cash transfer intervention program on rural left-behind children’s nutritional status and development in poor rural areas of China [13]. Hunan Province, located in Central South China, is one of the provinces where more than half of the resident children are left-behind children. We initially chose Fenghuang County and Anhua County as the sites, which include locations with geographic diversity and multiethnic populations for this project. Fenghuang County is an impoverished mountain region in west of Hunan, with over 60% of the population represented by minority groups. Anhua County is a typical plain region with 99% of the population of Han nationality. However, finally data collection did not take place in Anhua County because of limitations in local health manpower. We finally chose Pingjiang County which also has a high LBC population and has the similar geographic characters, economic levels, and ethnic composition to Anhua County.

### 2.2. Participants

Village inclusion criteria: villages were included if they had a minimum of 15 left-behind children (aged 3–5 years) living in poor households (defined as annual income < RMB 2300), had no kindergarten or care centers for LBC, and did not receive any funding or benefits in kind from other sources, such as charities or Non-Governmental Organizations (NGOs).

Participant inclusion criteria: All the 3–5-years-old LBC and their caregivers in the selected villages were eligible. Eligibility criteria for households in the intervention villages were as follows: (1) households which were caring for at least one left-behind child (3–5 years); (2) “poor households”, defined as average per-capita annual income lower than RMB 2300; (3) households which were not receiving benefits from other charities, NGOs or other similar programs.

### 2.3. CCT Interventions

The intervention began in May, 2015 and continued for one year. CCTs were used to incentivize caregivers to attend the nutrition education workshop and accompany the LBC to use basic public health services. Poor households with at least one eligible LBC, identified as beneficiaries for intervention, could receive RMB 500 (equal to USD 78.8) as a health grant (for LBC using basic public health services), and RMB 300 (equal to USD 47.3) as a nutritional education grant (for caregivers attending six nutrition workshops). A maximum of three LBC beneficiaries were covered by the health grant.

Workshop 1—Introduction of basic public health services for LBC
Workshop 2—Importance of health examination for LBC
Workshop 3—Importance of nutrition and diet diversity for LBC’s health
Workshop 4—Common nutrition problems for LBC (stunting)
Workshop 5—Common nutrition problems for LBC (Iron Deficiency Anemia, DA)
Workshop 6—Health evaluation and its importance for LBC

The health service cash transfer aimed to increase health service use for 3–5-year-old LBC. Households could receive RMB 200 (equal to USD 315.4) cash for each eligible LBC visiting the town hospital to receive annual regular growth monitoring (in 2015 and 2016), and RMB 100 (equal to USD 15.8) for age-appropriate vaccinations. Prior to the intervention, the town hospitals doctors received training in providing nutrition education for caregivers and management of CCT payment lists. The nutrition workshop for caregivers in the intervention group was held in town hospital once every two months. The caregivers in intervention group were notified by a phone call about the time, location, and content of workshop two weeks in advance. The caregivers attended were registered with conditional cash transfers. Colorful posters and flyers were provided by research group. Left-behind children and the caregivers in the control group received the health service as usual during the intervention period. The caregivers were also able to participate in free health education as usual in town hospitals, which were irregularly scheduled and without cash incentives.

### 2.4. Outcome Measures

Face-to-face questionnaire interviews were carried out before and after the intervention, between January and March 2015 and between June and July 2016. Caregivers’ nutrition knowledge and food practices with respect to LBC were investigated for both the control and intervention group. The knowledge of nutrition was assessed using an eight-item questionnaires. The caregivers were asked whether they were aware of the importance of children’s height and weight, whether they knew that when a child is not hungry this does not necessarily mean he/she is obtaining enough nutrients, whether they knew eggs and dairy products were good food choices for children, whether they knew that skipping breakfast was not good for children, whether they recognized the symptoms of iron deficiency anemia (pale skin, weakness, dizziness, headache, etc.) and whether they were able to identify iron-rich food resources (can give examples, like liver, beef, pork, and fish, etc.). Each correct response was given a point of 1 and a wrong response or a “unclear” response a point of 0. The knowledge score range was 0 to 8. Low dietary diversity is a predictor of child malnutrition and stunting. In the pilot household survey, we found some rural poor left-behind families only had one dish for each meal. Diet quality is poor when only a small variety of food is available. Therefore, the food practice behaviors in last three months were evaluated using five behavioral items with a points system. These items were: frequency of preparing breakfast for the family; frequency of preparing a separate protein-rich meal for the child (meat, fish, etc.); frequency of preparing two dishes or more for each meal; frequency of preparing eggs for the child; and frequency of preparing dairy products for the child. The answer choices were “less than 2 times a week” (1 point), “3–4 times per week” (2 points), and “5–7 times per week” (3 points). The food practice score range was 5 to 15.

General characteristics of CLBC and households were also investigated.

### 2.5. Sample Size

Initial calculations indicated that a sample size of 600 left-behind children (15 left-behind children in each village) was needed to detect a significant difference in the primary study outcome, which was the difference in IDA prevalence. Allowing for a 33% drop-out rate during the course of study, the evaluable number of left-behind children would be 200 in each group. The prevalence of IDA among under-fives in rural Hunan was reported as 34.3% in 2011 [14]. Assuming a conservative estimate for the interclass correlation co-efficient of 0.01 and at 5% significance, we would have 80% or more power to detect differences of 13% or greater in the primary outcome with this sample size (i.e., from 34 to 21%).

### 2.6. Randomization

In 2014, the socioeconomic information and characteristics of 3–5-year-old LBC and their families in 277 villages in Pingjiang County and 340 villages in Fenghuang County were provided by local governmental departments. A total of 72 villages in Pingjiang County and 132 villages in Fenghuang County met the village criteria as the sample pool. Using a cluster randomized controlled trial (CRCT), 40 villages were randomly selected. A total of 15 LBC aged 3–5 years old and their CLBC were randomly selected from the village.

Villages were the unit of randomization. We stratified villages according to the income level, number of population, number of left-behind children and distance to town hospital. After baseline investigation, allocation concealment was achieved by undertaking all baseline measures. A blocked balancing algorithm was used to randomize villages to either the intervention or control arm [15]. The randomized villages contained a nearly equal number 3–5-year-old left-behind children and the allocation designs minimized the imbalance between covariate means. The covariates included within this algorithm included number of 3–5-year-old LBC in the village, average annual household income, distance between the village and town hospital, ethnicity, use of basic health service among left-behind children in the previous year, and IDA prevalence. Once two villages allocated to different arms were found to be too close to each other (less than 5 km), the random sequence generator was re-run to avoid contamination.

### 2.7. Participant Recruitment

This study provided conditional cash transfers to rural poor households with 3–5-year-old left-behind children. Eligible study subjects were identified by the research group with the assistance of local village doctors. All the eligible left-behind children and the main caregivers were registered by village doctors prior to investigation. A total of 15 eligible left-behind children and the main eligible caregivers from each household were randomly selected from the lists and invited to participate in the study. If the siblings of the eligible LBC met the criteria, she or he was also invited to participate in the study. The research group explained the goal of the study, the procedure, and the risks and benefits to the caregivers. The left-behind children and caregivers were registered after agreeing to be involved in the study with signed informed consent. The intervention has been implemented for one year. The caregivers participated in the questionnaires surveys in the village clinics through a phone call notice from the village doctors, before and at the end of the intervention. Every caregiver was offered a reimbursement of RMB 60 to cover their transportation cost at both time points.

### 2.8. Ethical Approval

This research was approved by the independent ethics committee of the Institute of Clinical Pharmacology, Central South University (ctxy-140003) and was registered in the China Clinical Trial Register (ChiCTR-TRC-14005117). During the investigation, informed consent was obtained from all participants prior to questionnaire administration.

### 2.9. Statistical Analysis

EpiData 3.0 software (The EpiData Association, Odense, Denmark) was used for data entry. Data cleaning, random number generation, and analysis were conducted by IBM SPSS 18.0 software package (IBM Corp., Armonk, NY, USA). All the outcomes were analyzed before and at the end of intervention. The statistical methods used in this research include statistical descriptions and chi-squared tests. A generalized linear mixed model (GLMM) was used to examine the effect of the conditional cash transfer on the caregivers’ nutritional knowledge and their food practices with respect to LBC.

## 3. Results

**Figure 1:**
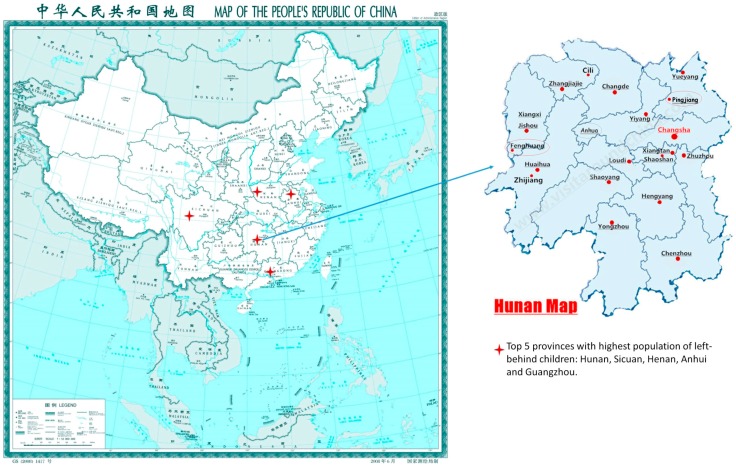
Location map of the sampling sites.

In the baseline investigation, consent was obtained from 518 caregivers in 40 eligible villages. At the end of the intervention, a total 447 caregivers were included in analysis (see Figure 1).

### 3.1. General Characteristics of Participants

Table 1 shows the baseline characteristics of CLBC and their families. The majority of the caregivers surveyed were female (>60%) and farmers (>90%). Approximately 30% of caregivers had no formal education and more than 80% were grandparents of the LBC. There were no significant demographic differences between the intervention group and the control group before or after the intervention.

### 3.2. Knowledge of Nutrition and Food Practices Related to Children among CLBC

**Figure 2:**
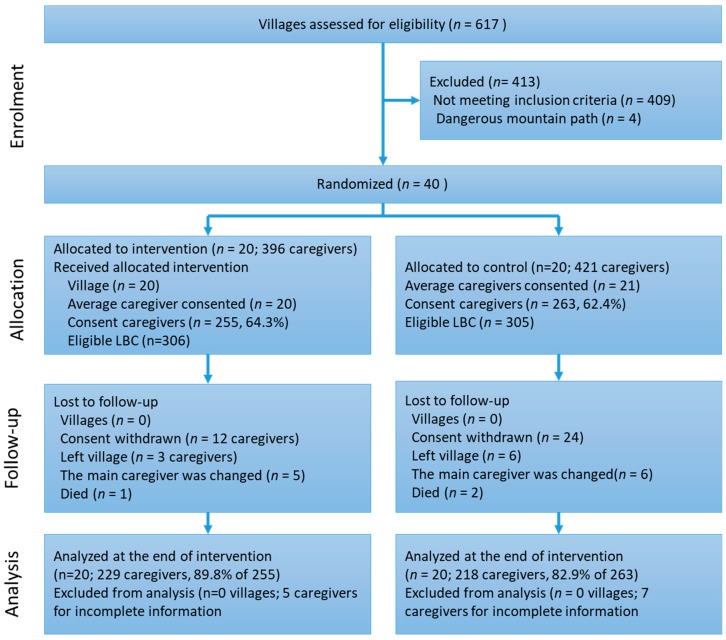
Flow of caregivers’ recruitment and trial allocation

During the intervention period, 26 caregivers in intervention group and 45 in control group dropped out (see Figure 2). After the intervention, intervention group CLBC scores for timely understanding of height and weight of LBC, food diversity, egg and dairy choices, and anemia identification and prevention were significantly higher than those of the control group (*p* < 0.05), as shown in Table 2.

According to Table 3, after the intervention, there was a significant difference in food practices relating to dairy products (*p* = 0.034) and eggs (*p* = 0.040) between the intervention and control groups of CLBC. However, in terms of the proportion of intervention group and control group CLBC who prepared breakfast for the children, who prepared a separate meal for the children, and who prepared meals with more than two dishes, there were no statistical differences.

### 3.3. The Effectiveness of the Intervention on Nutrition Knowledge and Food Practice Behaviors

**Figure 3:**
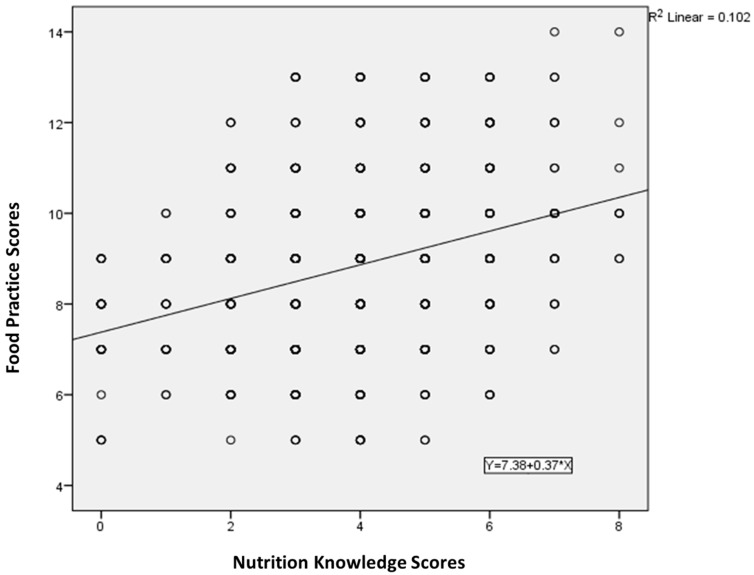
Relationship between nutritional knowledge score and food practice score, based on linear regression (F = 101.813, *p* < 0.01).

A generalized linear mixed model was used to evaluate the effect of the CCT intervention on caregivers’ nutrition knowledge score and food practice score over time in Table 4. The LBC was included as a random effect. The GLMM had seven fixed predictors: the group (0 = control, 1 = intervention), period (0 = baseline, 1 = endpoint), age (in years), gender (0 = female, 1 = male), ethnicity (0 = Han nationality, 1 = minority), education level (1 = no education, 2 = less than 3 years, 3 = 3–6 years, 4 = middle school, 5 = high school), and socioeconomic level (1 = low, 2 = middle, 3 = high). The intervention proved to be effective on subjective nutritional knowledge (*p* = 0.01). The significant intervention effect concerning food practice scores was also confirmed (*p* = 0.047). Figure 3 showed that there was a positive correlation between the nutritional knowledge scores and food practice scores of caregivers (R^2^ = 0.102, *p* < 0.01).

## 4. Discussion

The baseline survey found that the caregivers of left-behind children (CLBC) in rural, poverty-stricken areas of Hunan Province had poor nutritional knowledge and unreasonable dietary structure arrangements for their children. Although most CLBC recognize that “eating a variety food is good for the child” (82.1%) and that “it is important to know the child’s height and weight” (76.1%), there are still many CLBC who think that “When child is not hungry this means he/she gets enough nutrients.” (82.3%) and that “Skipping breakfast is not good for the child” (68.2%). This is similar to other studies [16,17,18].

Nutritional knowledge is the driving force of behavior change, and positive nutrition knowledge can guide individuals to adopt correct diet behavior. The results show that rural CLBC undertake unreasonable food choices and food practices for children. This population has limited financial means, and food is scarce. A food frequency questionnaire found that many families can eat pork only once a month and chicken only once a year. In addition, there were ethnic differences in food practices, and the family diet for the Han population was significantly better than for ethnic minorities (F = 7.985, *p* = 0.008). Many Miao/Tujia families traditionally only eat two meals a day and do not pay much attention to breakfast. Such caregivers believe that it is enough as long as children feel full, and that if they do not feel full at one meal, they can simply eat more at the next. This concept has been passed down through generations and is difficult to change in a short period of time. In this study, the Tujia and Miao ethnic minority LBC were mainly distributed in the poor Xiangxi mountainous areas, while the Han LBC were mainly distributed in the hilly areas of northeastern Hunan. The regional differences were obvious. The Xiangxi mountainous area has a weaker economy, more underdeveloped agriculture, and more inconvenient transportation than the hilly areas. Food is scarcer, with grain vegetables and beans mostly available only from personal family plots, and there is a highly inadequate animal product intake. Therefore, the caregiver lacks the objective conditions to arrange reasonable and diverse daily meals for LBC. Secondly, the primary caregivers of LBCs are mostly grandparents with their own issues to contend with, such as geriatric disease and limited economic resources. When they shop for food, they pay the most attention to food prices, even if they know what the more nutritious food options are. This suggests that LBC nutrition policies should take into account different ethnic customs, regional economic levels, and family incomes to maximize the effectiveness of intervention measures.

Many poor rural areas in China have no kindergarten or centralized care agencies, and on account of the unique family situations of LBC, nutrition improvement policies for this group are often difficult to implement. At present, there is no nutrition intervention policy or project for 3–5-years-old LBC in rural areas. CCT programs are seen as effective ways to reduce inequality, break the vicious circle of intergenerational transmission of poverty, and improve the health of women and children, including nutritional status and school enrollment rates, around the world [19,20,21]. We found that CCTs, in combination with nutritional education, could improve the nutritional knowledge and the food practice behaviors of CLBC to a certain extent. Even though health education is included in Chinese primary health care, the resources to receive health knowledge are limited. In our study, no doctor had received nutritional training before and less than a fifth of caregivers had participated in health education workshop organized by local health institutions [4]. Lack of transport facilities and cost of transport were the barriers for caregivers to access health services, including health education. The incentives of cash transfer could promote caregivers to attend the nutrition education workshop. Our intervention significantly increased the nutritional knowledge of CLBC. In interviews, we also found that CLBC tended to increase the consumption of relatively expensive animal food products after accepting nutritional education and receiving cash subsidies. After the intervention, the knowledge received helped CLBC improve their food choices and food practices, especially in terms of the frequency of egg and milk provisions for children. However, the correlation between knowledge and practice was not strong, with R^2^ = 0.102. Food purchasing and food choices were influenced by other factors, e.g., household economic status, local food sources, etc. On the other hand, cash transfers allowed caregivers to purchase better foods in greater quantities. Generalized linear mixed model analysis further showed that in our study the CCT intervention was associated with a better nutritional knowledge level (adjusted *p*-value = 0.01) and better food practice behaviors (adjusted *p*-value = 0.047) over time, compared with baseline. It is noteworthy that although the CCT intervention in our study did improve the food practices of CLBC with respect to their children, overall LBC dietary structure remained subpar. For example, the “Chinese 0 to 6-year-old children’s dietary guidelines” stipulate that preschool children should drink milk every day, but in our study after the intervention, only 13.2% of the intervention group LBC reached the level of drinking milk 5 to 7 times a week [22]. Because of the size of the preschool-age population in rural China, low family incomes, and insufficient dietary nutrient intake, anemia and growth retardation are prominent problems [23,24]. A single CCT intervention may have limited effectiveness in improving dietary intake, but intervention effectiveness can be maximized by combining CCTs with other programs that target factors such as regional economic status, food resources, and social support.

This study is the first to report the impact of CCT intervention on nutritional knowledge and food practices among the caregivers of disadvantaged LBC in rural China. Our results will also provide information for future development of relevant health policies to reduce inequities in healthcare. However, there are a couple limitations. As the follow-up time was only one year, the long-term effect of CCT interventions has not been evaluated. In addition, most of the caregivers interviewed were elderly citizens with low education levels and many were ethnic minorities, so deviations in the questionnaires may exist due to multiple translations. The sample size for the whole project was calculated by assuming a 13% difference between groups for the primary outcome, the prevalence of IDA in LBC. However, the subjects of this article were the caregivers and the difference in terms of average correct nutritional knowledge between the groups was 11%. Subsequent studies and larger sample sizes are necessary for follow-up assessments of CCT interventions targeting the nutritional and health development of low-income children, as well as cost-effectiveness analyses. 

## 5. Conclusions

The CCT intervention in this study improved the nutritional knowledge and positive food practice behaviors of the main caregivers of 3–5-year-old LBC in Hunan Province. There are many CCT programs for rural population, but conditional cash transfer combined with nutrition education may increase nutritional knowledge and promote more food practice changes. It is also important to consider the characteristics of rural LBC and their caregivers in health intervention programs. For these vulnerable populations, a variety of policies should be adopted to promote an improved nutritional status and healthy growth and development.

## Figures and Tables

**Table 1:** General characteristics of caregivers of left-behind children (CLBC) and households at baseline and the endpoint.

Variables	Baseline, January–March 2015 (*n* = 518)			Endpoint, June–July 2016 (*n* = 447)		
	Control (*n* = 263)	Intervention (*n* = 255)	*p*	Control (*n* = 218)	Intervention (*n* = 229)	*p*
Household Size	6.7 ± 2.2	6.8 ± 2.0	0.643	6.7 ± 2.1	6.8 ± 2.0	0.824
Female Caregivers (*n*, %)	165 (62.7%)	178 (69.8%)	0.095	143 (65.6%)	166 (72.5%)	0.125
Minorities (*n*, %)	95 (36.1%)	98 (38.4%)	0.325	80 (36.7%)	87 (38.0%)	0.427
Age of Caregivers (years)	54.3 ± 12.6	56.2 ± 12.0	0.075	55.9 ± 13.0	57.6 ± 12.4	0.172
Number of 3–5-year-old LBC being taken care of ^#^:						
1	205 (78.5%)	196 (77.5%)	0.856	174 (79.8%)	181 (79.0%)	0.979
2	48 (18.4%)	47 (18.6%)		35 (16.1%)	38 (16.6%)	
3 and above	8 (3.1%)	10 (4.0%)		9 (4.1%)	10 (4.4%)	
Education level of caregivers						
No education (*n*, %)	81 (30.9%)	74 (29.0%)	0.324	67 (30.7%)	73 (32.0%)	0.803
Less than 3 years (*n*, %)	47 (17.9%)	61 (23.9%)		43 (19.7%)	46 (20.2%)	
3–6 years (*n*, %)	74 (28.2%)	68 (26.7%)		64 (29.4%)	57 (25.0%)	
Middle school level (*n*, %)	52 (19.8%)	40 (15.7%)		37 (17.0%)	41 (18.0%)	
High school level (*n*, %)	8 (3.1%)	12 (4.7%)		7 (3.2%)	11 (4.8%)	
Caregiver–LBC relationship						
Mother (*n*, %)	29 (11.0%)	23 (9.0%)	0.560	23 (10.6%)	21 (9.2%)	0.636
Father (*n*, %)	12 (4.6%)	12 (4.7%)		8 (3.7%)	5 (2.2%)	
Grandparent (*n*, %)	211 (80.2%)	214 (83.9%)		180 (82.6%)	198 (86.5)	
Other (*n*, %)	11 (4.2%)	6 (2.4%)		7 (3.2%)	12 (2.7%)	
Caregiver career						
Unemployed	16 (6.1%)	14 (5.5%)	0.376	14 (6.4%)	10 (4.4%)	0.487
Farmer	239 (90.9%)	227 (89.0%)		199 (91.3%)	211 (92.1%)	
Employed	8 (3.0%)	14 (5.5%)		5 (2.3%)	8 (3.5%)	
Caregiver marital status						
Married	182(83.1%)	198 (86.8%)	0.615	178 (81.3%)	194 (85.1%)	0.514
Separated	3 (1.4%)	3 (1.3%)		7 (3.2%)	3 (1.3%)	
Divorced	6 (2.7%)	3 (1.3%)		5 (2.3%)	4 (1.8%)	
Widowed	28 (12.8%)	24 (10.5%)		29 (13.2%)	27 (11.8%)	
Socioeconomic status (tertiles) *						
Low	83 (31.6%)	89 (35.0%)	0.418	66 (30.3%)	80 (35.2%)	0.158
Middle	95 (36.1%)	78 (30.7%)		82 (37.6%)	66 (29.1%)	
High	85 (32.3%)	87 (34.3%)		70 (32.1%)	81 (35.7%)	
Number of LBC in the family	2.0 ± 1.0	2.1 ± 1.0	0.537	2.1 ± 1.1	2.2 ± 1.1	0.345

* Socioeconomic status, which was estimated following principal component analysis, including various items related to the economic status (family size, household annual income, size of land used for cultivation, housing type, access to tap water, and number of bedridden patients at home). ^#^ Missing data for baseline investigation. LBC: left-behind children.

**Table 2:** Comparison of nutritional knowledge between the intervention arm and the control arm of CLBC, at the end of the intervention.

Knowledge Item	Correct Responses (%)		*p*
	Control Group (*n* = 218)	Intervention Group (*n* = 229)	
K_1_: It is important to know the child’s height and weight.	144 (66.1%)	196 (85.6%)	<0.001
K_2_: When child is not hungry this means he/she gets enough nutrients.	37 (17.0%)	42 (18.3%)	0.712
K_3_: Skipping breakfast is not good for the child.	61 (28.0%)	81 (35.4%)	0.104
K_4_: Eating a variety of food is good for the child.	165 (75.7%)	209(88.2%)	0.001
K_5_: Eggs are a healthy food choice for the child.	167 (76.6%)	209 (91.3%)	<0.001
K_6_: Dairy products are a healthy food choice for the child.	150 (68.8%)	182 (79.5%)	0.013
K_7_: Is able to recognize the symptoms of iron deficiency anemia (pale skin, weakness, dizziness, headache, etc.)	49 (22.5%)	75 (32.8%)	0.02
K_8_: Is able to identify iron-rich food (can give examples, like liver, beef, pork, and fish, etc.).	19 (8.7%)	54 (23.6%)	<0.001

**Table 3:** Comparison of food practice behaviors between the intervention group and the control group of CLBC at the end of the intervention.

Variables	Control Group (*n* = 218)	Intervention Group (*n* = 229)	*p*
B_1_: Prepares breakfast for the family.			
Less than 2 times per week	22 (10.0%)	31 (13.6%)	0.149
3–4 times per week	18 (8.2%)	10 (4.4%)	
5–7 times per week	179 (81.7%)	187 (82.0%)	
B_2_: Prepares a separate meal for child (meat, fish, etc.)			
Less than 2 times per week	165 (75.3%)	162 (71.1%)	0.325
3–4 times per week	25 (11.4%)	24 (10.5%)	
5–7 times per week	29 (13.2%)	42 (18.4%)	
B_3_: Prepares two dishes or more for each meal.			
Less than 2 times per week	79 (36.1%)	64 (28.1%)	0.129
3–4 times per week	73 (33.3%)	77 (33.8%)	
5–7 times per week	67 (30.6%)	87 (38.2%)	
B_4_: Provides eggs for the child.			
Less than 2 times per week	146 (66.7%)	129 (56.6%)	0.040
3–4 times per week	44 (20.1%)	69 (30.3%)	
5–7 times per week	29 (13.2%)	30 (13.2%)	
B_5_: Provides dairy products for the child.			
Less than 2 times per week	159 (72.6%)	140 (61.4%)	0.034
3–4 times per week	35 (16.0%)	56 (24.6%)	
5–7 times per week	25 (11.4%)	32 (14.0%)	

**Table 4:** Results of effectiveness of the conditional cash transfer (CCT) intervention on nutrition knowledge and food practice scores for CLBC.

Variables	Baseline	Endpoint	*p*-Value	*p*-Value Adjusted ^ϕ^
**Nutrition Knowledge Score**				
Intervention (*n* = 228)	3.68 ± 1.43	4.47 ± 1.44	0.02 *	0.007 **
Control (*n* = 219)	3.72 ± 1.56	3.67 ± 1.73		
**Food Practice Score**				
Intervention (*n* = 228)	8.65 ± 1.85	9.35 ± 1.80	0.057	0.031 *
Control (*n* = 219)	8.54 ± 1.80	8.89 ± 1.95		

Notes: * *p* < 0.05, ** *p* < 0.01, generalized liner mixed model (GLMM) with gamma distribution for repeated measures. ^ϕ^ GLMM controlled for age, gender, race, education level and socioeconomic level.

## Abbreviations

The following abbreviations are used in this manuscript:

CLBC	Caregivers of left-behind children
LBC	Left-behind children
CCT	Conditional cash transfer
